# Food for thought

**DOI:** 10.3325/cmj.2012.53.77

**Published:** 2012-02

**Authors:** Gwinyai Masukume

**Affiliations:** 1 Department of Obstetrics and Gynecology, Mpilo Central Hospital, Bulawayo, Zimbabwe; 2 Cuisine Research Institute (CRI); For the GRAPE (Group for Research and Advancement of Palatable Eponyms) study team.

The history of eponyms in obstetrics and gynecology is rich and long (1). Eponyms are here to stay (2,3).

Distinguishing whether the entry of Sonic the Hedgehog, the video game, cartoon, comic strip, and film character along with his great nemesis, Dr Ivo Robotnik, into the medical world is frivolous or dignified is largely a matter of personal taste, as well as is the use of other eponyms in medicine (4-6). However, analogies are a very important aspect in various areas of life and the use of analogies in medicine is no exception. Food-related medical analogies, like other analogies, assist with naming, learning, remembering, reasoning, and consequently practice (7). Despite their importance, medical literature only occasionally deals with food-related medical metaphors. The beauty of these culinary medical terms lies in the fact that their use is generally idiosyncratic, random, and inconsistent, and is influenced by time, place, and culture (2).

Presented is an admittedly cherry-picked list of fascinating food-related medical terms in obstetrics and gynecology to whet the appetite (Table 1). We are only human and deserve some comic relief (34,35). Food for thought.

**Table 1:** Cherry-picked food-related medical metaphors in obstetrics and gynecology

Analogy	Class	Brief description
Almond-shaped ovaries	fruit	female gonads akin to almonds in shape (8)
Banana sign	fruit	shape of fetal cerebellum on ultrasound scan suggestive of spina bifida (9)
Bean-shaped G spot	legume	controversial erogenous zone located on anterior vaginal wall (10)
Cauliflower warts	vegetable	condyloma acuminata shape (11)
Chocolate cyst	confectionary	ovarian endometriosis, color of old blood (12)
Cone biopsy	dessert	cervical conisation for malignancy (13)
Cottage cheese appearance	dairy	thick white curds in vulvovaginal candidiasis similar to look of cheese (14)
Crab	sea food	pubic lice – *phthirus pubis* (15)
Fish flesh appearance	sea food	of leiomyosarcoma (16)
Fishy odor	sea food	fish odor in bacterial vaginosis due to trimethylamine (17)
Fourchette – little fork	utensil	fork-shaped posterior junction of labia minora (18)
Grape-like vesicles	fruit	molar pregnancy, trophoblastic tissue resembles grape clusters (19)
Lemon sign	fruit	concave frontal bones on ultrasound scan giving the fetal skull a lemon appearance, suggestive of spina bifida (20) (Figure 1)
Milk leg	dairy	*phlegmasia alba dolens*, more common in pregnancy (21)
Morula – mulberry	fruit	16-32 cell stage fertilized ovum (22)
Omental cake	confectionary	tumor infiltration of omentum classically arising from ovarian carcinoma - cake is visible on radiologic investigations (23)
Peach fuzz in anorexia nervosa	fruit	serious eating disorder commonly affecting young women (24)
Pear-shaped uterus	fruit	pear-shaped internal sex organ (8)
Placenta – flat cake	confectionary	disc-shaped organ, name derived from Latin root for a flat cake (25)
Port-wine amniotic fluid	drink	color of amniotic fluid sometimes found with placental abruption (26)
Sarcoma botryoides	fruit	malignant tumor which may affect the vagina usually in children and is grape-shaped (botryoid) (27)
Strawberry cervix	fruit	cervical bleeding in spotted pattern due to trichomoniasis (28)
Strawberry-shaped skull	fruit	sonographic marker of serious fetal abnormality such as trisomy 18 (29)
Swiss-cheese endometrium	dairy	cystic endometrial hyperplasia (30)
Waiter's tip deformity	serve food	brachial plexus upper trunk lesion because of difficult delivery (31)
Wharton's jelly	dessert	Wharton's jelly of the umbilical cord which is gelatinous (32)

**Figure 1:**
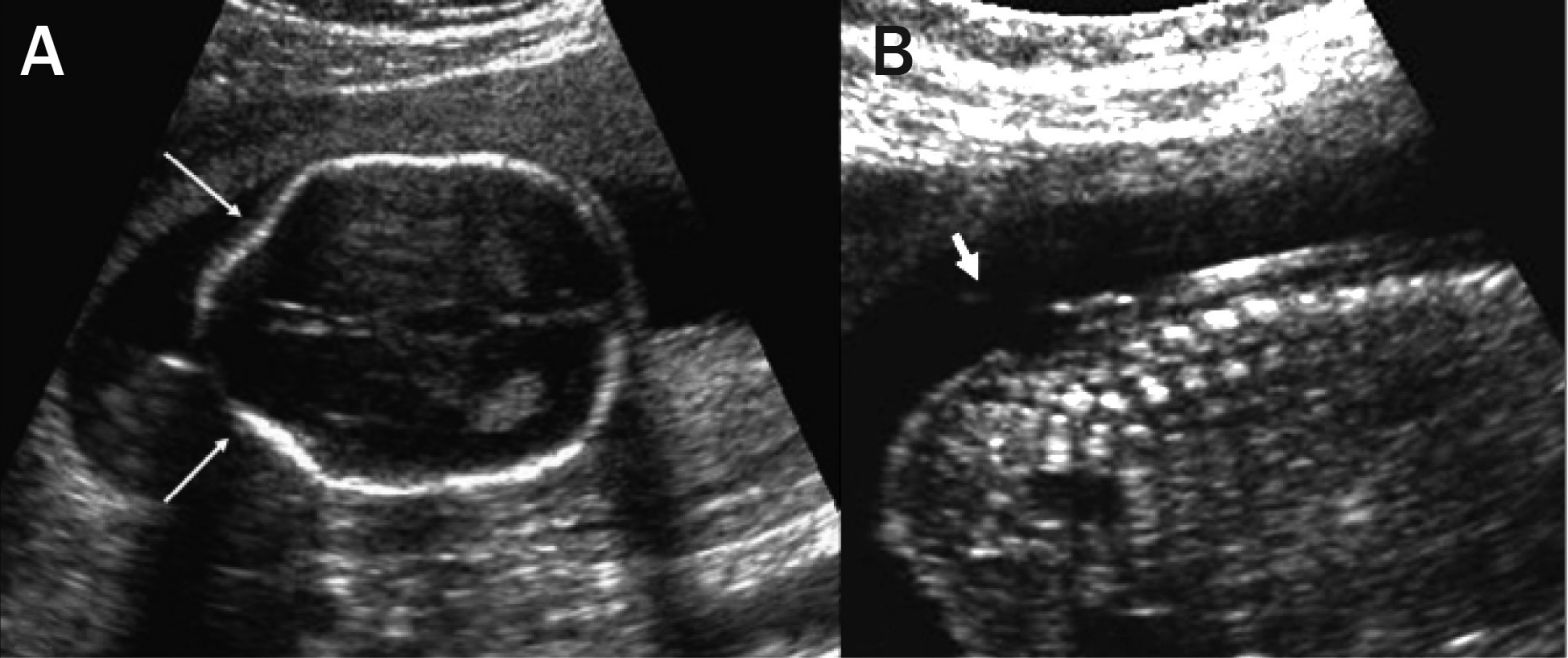
Lemon sign. Axial sonographic image of the fetal head at the level of the biparietal diameter (**A**) shows inward concavity of both frontal bones (arrows) instead of the normally seen convexity. This gives the head the appearance of a lemon. Sagittal sonographic image of the fetus (**B**) shows a meningomyelocele (thick arrow) in the lumbar region. Reproduced with kind permission of the Indian Journal of Radiology and Imaging (33).
